# Power and Performance Management in Nonlinear Virtualized Computing Systems via Predictive Control

**DOI:** 10.1371/journal.pone.0134017

**Published:** 2015-07-30

**Authors:** Chengjian Wen, Yifen Mu

**Affiliations:** 1 Department of Computer Science and Engineering, Beihang University, Beijing, China; 2 Key Laboratory of Systems and Control, Institute of Systems Sciences, Academy of Mathematics and Systems Science, Chinese Academy of Sciences, Beijing, China; Nankai University, CHINA

## Abstract

The problem of power and performance management captures growing research interest in both academic and industrial field. Virtulization, as an advanced technology to conserve energy, has become basic architecture for most data centers. Accordingly, more sophisticated and finer control are desired in virtualized computing systems, where multiple types of control actions exist as well as time delay effect, which make it complicated to formulate and solve the problem. Furthermore, because of improvement on chips and reduction of idle power, power consumption in modern machines shows significant nonlinearity, making linear power models(which is commonly adopted in previous work) no longer suitable. To deal with this, we build a discrete system state model, in which all control actions and time delay effect are included by state transition and performance and power can be defined on each state. Then, we design the predictive controller, via which the quadratic cost function integrating performance and power can be dynamically optimized. Experiment results show the effectiveness of the controller. By choosing a moderate weight, a good balance can be achieved between performance and power: 99.76% requirements can be dealt with and power consumption can be saved by 33% comparing to the case with open loop controller.

## 1 Introduction

In large scale computing systems, like data centers, huge power has been consumed and the consumption is increasing greatly each year([1]). This gives rise to heavy pressure on environment and resource. Thus performance and power management has become more and more important and captured growing research attention in both industrial and academic fields.

As an advanced technology, virtualization can provide a promising approach to save power ([2]) and has become a basic architecture in most data centers today. In the virtualized systems, the services are enclosed in the virtual machines (VM). Many VMs can be hosted on one physical machine (PM) and the VMs can be created or destroyed or migrated between different PMs at little cost. Then, by consolidating many VMs to few PMs, the PMs with low workload, utilization or frequency can be shut down and power consumption can be reduced. Hence, in the virtualized computing systems, more control actions is feasible, including turning on/off the PMs, turning on/off the VMs, migrating the VMs between PMs, regulating the frequency of processor etc.. Additionally, control actions often have time delay. For example, turning on/off the PM needs several minutes and thus will influence performance and power consumption greatly. These factors make it complicated to design sophisticated and fine control to manage performance and power consumption.

Because of the significance and difficulty, researchers have been trying to solve the problem from different aspects. Usually, the first step is to build a model of energy consumption(called power model below for simplicity). In the literature, linear power model is often adopted. However, modern machines show significantly nonlinear features, which can be caught from data(see Section 2). Other modelling include fuzzy model etc., see e.g. [3, 4]. A review for power metering can be seen in [5]. With power model, the problem can be formulated in several ways. For example, we can maximize performance under power budget(e.g. [6]) or minimize power consumption when tracking performance or load balance between VMs (e.g. [7] ), or optimize a newly defined objective, which integrates performance, power and the balance between different machines(e.g. [3][8]).

Based on the formulations, different kinds of solutions have been proposed, see [9] for a review of energy-efficient algorithms. Next we describe several specific ways. In [3][10], the authors use reinforcement learning, and in [3] the reinforcement learning method is used together with fuzzy rule bases to achieve a defined objective, which shows robust performance improvement. In [11], the authors take advantage of the min-max and shares features inherent in virtualization technologies to allocate resources to a VM based on available resources, power costs, and application utilities. In [12], a temperature-aware workload placement is proposed in data centers. In [13], game theory is applied to formulate the problem and optimize power and performance at each level of the hierarchy while maintaining scalability.

Since it can provide a unified framework as well as rigorous controller design and can deal with dynamic and uncertain environment, control theory has been applied more and more to solve the problem. For example, in [7], the self-tuning regulator(which is an adaptive controller) is utilized to track performance and then optimize the energy assumption based on linear power model; in [8] the authors designs optimal controller by integrating the SLA function, and introduces a two-level control with one level being faster and the other being slower; in [14] Kalman filter is introduced to track the CPU utilizations and update the allocations of CPU resources to VMs accordingly; in [15] PID controller is proposed to manage power consumption and CPU utilization; in [16] MPC controller is designed; in [6] both PID controller and MPC are adopted at the same time; in [17] the authors compare effects of different controllers and find that predictive controller performs better and has some self-learning behavior. Other recent related papers are referred to [18–22].

This paper aims to provide a predictive controller for the virtualized computing systems with significantly nonlinear features on power consumption. First, significant nonlinearity on energy consumption of newer servers are discovered from data. Especially, we find that the max utilization/workload does not correspond to the max performance-power ratio. This announces invalidation of linear power model, which was commonly adopted in the literature. To deal with the nonlinear features, we build a discrete system state model as well as the state transition graph. Then, performance and power, especially power, is defined on each state, which is more accurate than linear power model. Furthermore, turning on/off a PM becomes feasible control actions, and the time-delay effect can also be taken into account. Based on the discrete model, we design a predictive controller naturally and minimize the quadratic cost function (which integrates both performance and power) dynamically by searching on the feasible state space. During the control process, the time-varying workload is predicted using an AR model. By choosing a moderate prediction horizon, the search approach becomes feasible. Experiment results validate effectiveness of the controller: by choosing a moderate weight, performance requirement can be satisfied for more than 99% times; meanwhile, the power consumption can be saved by 33% compared to the system without predictive controller. Part of this paper is presented in [23].

The paper is organized as follows. After a brief review on power models, Section 2 shows novel features on energy consumption in modern machines, which make linear power model not suitable any more. Section 3 formulates the problem by building a discrete state model. Section 4 describes the design of predictive controller. Section 5 validates the effectiveness of the controller by experiments. And finally, Section 6 concludes the paper with some remarks.

## 2 Novel Features on Power Consumption

### 2.1 Linear Power Models in the Literature

In order to save power, a suitable power model, which catches the relationship between energy consumption per time unit (power, *kilo* ⋅ *hour*)) and physical parameters when running the machine, needs to be built. In the literature, linear power model is commonly adopted for simplicity and convenience, in which power consumption is modeled as a linear function with respect to certain system parameters, like CPU utilization, CPU frequency or number of VMs. Below we give some typical examples.

In [6], the VM’s power *P*_*VM*_ is assumed to be *P*_*VM*_ = *c*_*freq*_ * *u*_*cpu*_, where *P*_*VM*_ denotes VM’s power, *u*_*cpu*_ is CPU utilization of the VM, and *c*_*freq*_ is a model parameter which is dependent on processor frequency.

In [8], the power (*kilo* ⋅ *hour*) is assumed to be *Power* = *P*_*idle*_ + *α* ⋅ *U*_*cpu*_, where *P*_*idle*_ denotes the power consumption at idle state, *U*_*cpu*_ denotes CPU utilization and *α* is a parameter which is dependent on the specific machine and application.

In [24], the speed(or frequency) of the server *s*(*GHz*) is assumed to be *s* = *s*_*b*_ + *α*(*P* − *b*), where *s*_*b*_(Hertz) denotes the speed of a fully utilized server running at *b* Watts, *P* (Watts) denotes the power allocated to the server, while the coefficient *α* (units of *GHz* *per* *Watt*) is the slope of the power-to-frequency curve, which can be obtained by experiment.

In [25], the processor power is modeled as an approximately linear function with respect to the DVFS level within the limited DVFS adaptation range available in real multi-core processors, i.e., *cp*(*k*) = *a*(*f*(*k* + 1) − *f*(*k*)) + *cp*(*k* − 1), in which *cp*(*k*) denotes power consumption of the entire chip in the *k*-th control period, *f*(*k*) denotes total aggregated frequency of all cores on the chip in the *k*th control period and *a* denotes a generalized parameter that may vary for different chips and applications.

**Fig 1:**
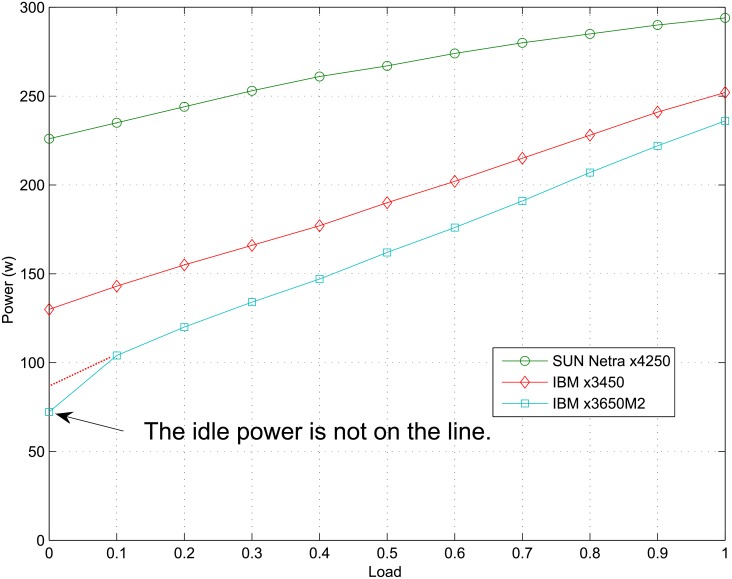
Linear model cannot reflect the reduced idle power of new machine.

In most practical cases, linear power model is suitable and can provide convenience for utilization, see [8]. This causes nonlinear power models rare to be studied in the literature. However, linear power model has a defect, which can be seen from Fig 1. For the server Sun Netra x4250 and IBM x3450, power can be approximated by a linear function with respect to CPU load, where the positive power value when load equals zero denotes the idle power. However, when the machine is turned off, the system load is also zero, but the power value now is zero rather than idle power. Thus, linear power model cannot distinguish the state when the PM is shut down from the state when it holds zero workload, which makes turning on/off a PM deleted from the optional control action set in most previous work. As we have stated in Section 1, this does not fit the virtualized computing systems.

### 2.2 Nonlinear Features of New Machines

In recent years, server technology has developed swiftly, making processors more and more advanced. Along with the progress, linear power model encounters new problems, making it unable to reflect novel features of new machines, as stated below.

First, a significant progress is the considerable reduction of idle power. Meanwhile, other parameters, such as power at big load, do not change with it. This makes linear power model no longer accurate, as shown in Fig 1.

In Fig 1, power curves for the servers Sun Netra x4250 and IBM x3450 are basically in accordance with a line. However, for the server IBM x3650M2, the idle power is reduced significantly (from 86 to 75 in fact), which is far below the line while other points still fit with it. Apparently, linear power model is no longer suitable now and thus management based on linear model is no longer optimal.

On the other hand, power saving is far from being the sole objective or even a prior objective. A good performance-power ratio(performance obtained per unit of power) makes more sense, since in practice it is more important to complete a job in the given time or satisfying other requirements.

From this point of view, in a linear power model, the performance-power ratio peaks at 100% load: assume

$$\begin{array}{c} \mathrm{Power}=P_{\mathrm{idle}}+\alpha \cdot Load, \end{array}$$

then the performance-power ratio

$$\begin{array}{c} \frac{\mathrm{Perf}}{\mathrm{Power}}=:\frac{\mathrm{Load}}{\mathrm{Power}}=\frac{1}{\alpha +\frac{P_{\mathrm{idle}}}{\mathrm{Load}}} \end{array}$$

is a strictly increasing function with respect to *Load* and peaks when *Load* = 100%.

**Fig 2:**
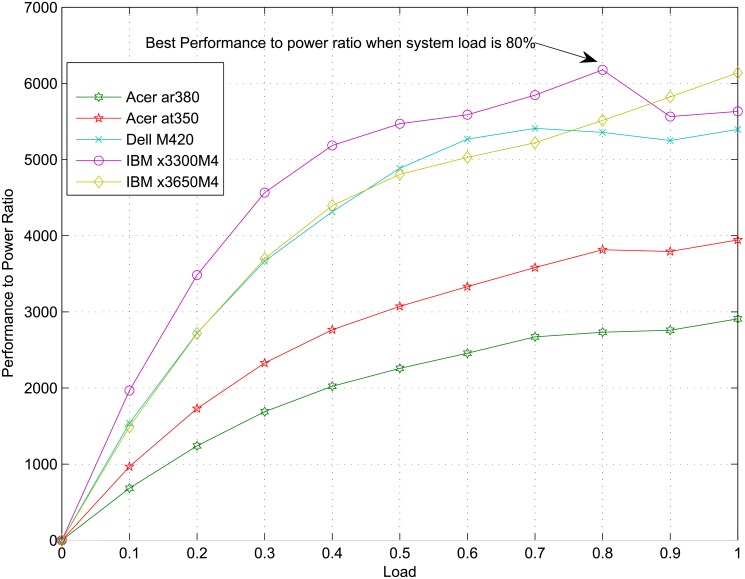
Performance-power ratio not peaks at 100% load.

However, the real case is different for new servers, as shown in Fig 2 below. Fig 2 draws the curve of performance-power ratio(defined as the requirements number that can be dealt with using one unit of power) with respect to load for the server IBM x3300M4 (data come from [26]).

From Fig 2, we can see that the biggest performance-power ratio occurs when the load is nearly 80% for some servers. Apparently, linear power model is not suitable any more and if we still use linear power model to simulate a modern server, we will miss the best choice. To deal with this, we will build a discrete-state-based power model in next section, which can include these nonlinear features.

## 3 Discrete State Model

In a virtualized computing system, there are some physical machines(PMs), denoted by *PM*_*i*_, *i* = 1, 2, …, *n*, which are homogeneous or heterogeneous. Virtual machines(VMs) are hosted on each PM and can be immigrated between different PMs. In this paper, we assume that at most one VM can be hosted on one processor of a PM.

Generally, the state of a PM describes whether the PM is turned on or off, the running frequency of its processors, and the VM number hosted on it. Thus, we define the state of *PM*_*i*_ as

$$\begin{array}{c} S_{i}=\left[on,\;vms,\;freq\right], \end{array}$$

in which the variable *on* is taken as *on* or *off*, corresponding to *PM*_*i*_ being turned on or turned off, *vms* stands for the number of VMs running on *PM*_*i*_, and *freq* represents the current frequencies of the running processors(so *freq* here is not a scalar).

In most situations, jobs allocated to one PM has the same type. Then, all influential factors are included in the state vector of a PM to complete the job. When different jobs are allocated to a PM, more factors, such as the architecture among the cores, must be considered, which is beyond the focus of this paper.

The time varying strength of a job is called *workload* and denoted by *λ*(*k*) at time *k*. Suppose at time *k*, the state is *S*_*i*_(*k*), then the performance of *PM*_*i*_, denoted by *perf*_*i*_(*k*), is defined to be the *maximal* *workload* which can be dealt with by *PM*_*i*_. Obviously, if the allocated computing resource is not sufficient, it can happen that *perf*_*i*_(*k*) < *λ*(*k*). Here we assume that a requirement will be abandoned if it cannot be dealt with immediately, i.e., no aggregation effect exists here.

On the other hand, at time *k*, the power consumption of *PM*_*i*_, denoted by *power*_*i*_(*k*), also is mainly determined by *S*_*i*_(*k*). In the experiment, *power*_*i*_(*k*) can be measured at each time.

Apparently, there is a tradeoff between high performance and low power consumption. Thus in practice, we usually aim to get a good balance between them. To this end, we will build a discrete state model of the system and then design predictive controller.

**Fig 3:**
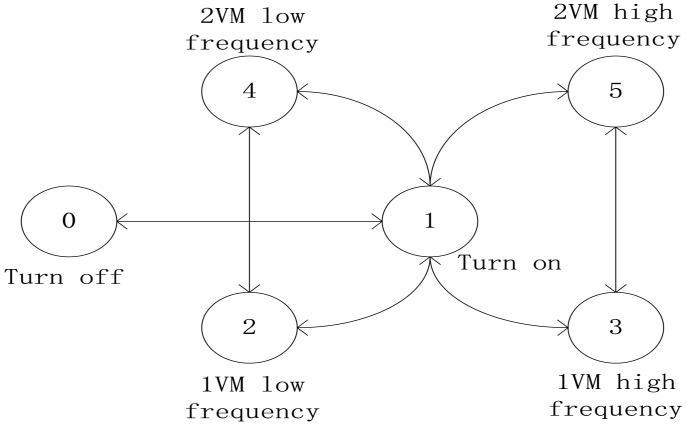
State transition graph for a single PM.

To simplify but without loss of generality, we describe the discrete model as we do in experiment. Let For *PM*_*i*_, *S*_*i*_ = [*on*, *vms*, *freq*] to be discrete and taken out of the following 6 typical values:

$$\begin{array}{l} s^{0}=\left[off,0,null\right],s^{1}=\left[on,0,null\right],\\ s^{2}=\left[on,1,1VM\;on\;low\;frequency\right],s^{3}=\left[on,1,1VM\;on\;high\;frequency\right],\\ s^{4}=\left[on,2,2VMs\;both\;on\;low\;frequency\right],s^{5}=\left[on,2,2VMs\;both\;on\;high\;frequency\right] \end{array}$$

which are abbreviated as **Turn off**, **Turn on, 1VM low frequency, 1VM high frequency, 2VM low frequency, 2VM high frequency** respectively. Then, by regulating resource, one state can be transformed to another, as shown in Fig 3, in which each directed line represents a transition between the states with positive time delay. Transition without time delay, such as the transition between *s* ^2^ and *s* ^3^, between *s* ^4^ and *s* ^5^, are omitted in the figure.

Now, assume that a job *J* is hosted on some PMs. Then, the performance and power at time *k* is defined on the state as

$$perfiJ_{i}^{J}\left(k\right)\;=\;function\left(S_{i}\left(k\right)\right)\in \left\{f^{J}\left(s^{q}\right),q=0,1,\ldots ,5\right\},$$ (1)

$$poweriJ_{i}^{J}\left(k\right)\;=\;function\left(S_{i}\left(k\right)\right)\in \left\{g^{J}\left(s^{q}\right),q=0,1,\ldots ,5\right\}.$$ (2)

In practice, the value of $perf_{i}^{J}(k),power_{i}^{J}(k)$ can be learnt by prior experiments.

For a system with *n* PMs, define the performance and power at time *k* as

$$perf\left(k\right)\;=\sum_{i=1}^{n}perf_{i}\left(k\right),$$ (3)

$$power\left(k\right)\;=\;\sum_{i=1}^{n}power_{i}\left(k\right),$$ (4)

which are reasonable in view of the meanings of *perf*_*i*_(*k*) and *power*_*i*_(*k*).

There are several advantages of the discrete state model

1. By defining discrete states and transition between them, turning on/off a PM can be treated as a control action. To be specific, *s* ^0^ to *s* ^1^ stands for turning on the machine and the reverse stands for turning it off.
2. The idle state is also included in the model by state *s* ^1^, and thus it can be taken into account. Accordingly, the performance-power ratio is hidden in the model naturally and can be reflected in the cost function. Thus the discrete state model overcomes the drawbacks of linear power model and is suitable for new servers.
3. Time delay effect, which exists during turning on/off a PM, now is taken into consideration. Time delay effect were often neglected in the literature, partly because it is hard to add into the model, partly because the PMs are always turned on in the past. Here, by modelling the system state discretely, time delay can be studied in a straight way.

In fact, the state transition graph builds a Markovian chain between states. This property, together with time delay effect, leads us to design predictive controller to regulate resources.

Finally, we note that this model is a coarse approximation to the real nonlinear relationship between performance, power and the system resource allocation. Putting it in a broader background, the method is commonly used to deal with a significantly nonlinear function. In the future, it is necessary and possible to make fine solution in which the frequency of each processor, the share of each virtual CPU, the memory and computing load will be added into the state.

## 4 Model based Predictive Controller

### 4.1 Controller Design

As we reviewed in Section 1, performance and power management problem can be handled in different ways: we can minimize power consumption under performance requirement, or we can maximize the performance within energy budget. Here we will define a quadratic cost function which combines performance and power together. By optimizing the cost function, we try to get a good balance between performance and power. To take time delay effect into consideration, the cost function must cover a time interval, which acquires the controller to predict the state for several time steps. The model predictive controller can just fit our scenario, see [24][25] for more introduction on the theory and applications of MPC.

Define the cost function at time *k* as below:

$$\begin{array}{c} cost(k)\;=\;cost(S(k),u(k\;+\;1),\;\ldots ,\;u(k\;+\;P))\\ \;≜\sum_{j=1}^{P}∥perf\left(k\;+\;j|k\right)\;-\;\hat{\lambda }(k+j){∥}_{Q_{j}}^{2}\;+\;\sum_{j=1}^{M}∥power\left(k\;+\;j|k\right){∥}_{R_{j}}^{2}\\ ≜\sum_{j=1}^{P}∥perf\left(\hat{S}\;+\;(k\;+j)|S(k)\right)\;-\;\hat{\lambda }(k+j){∥}_{Q_{j}}^{2}\;+\;\sum_{j=1}^{M}∥power\left(\hat{S}\;+\;(k\;+j)|S(k)\right){∥}_{R_{j}}^{2} \end{array}$$ (5)

where *j* = 1, …*P*, *P* > 0 is prediction horizon, 0 < *M* ≤ *P* is control horizon, *u*(*k* + *j*) is a feasible control action at time *k* + *j*; $\hat{\lambda }(k+j)$ is predicted workload at time *k* + *j*; $\hat{S}(k+j)$ is predicted state at time *k* + *j* according to Fig 3 if the control actions are take as *u*(*k* + 1), …, *u*(*k* + *j*) sequentially from the state *S*(*k*); *perf*(*k* + *j*∣*k*) and *power*(*k* + *j*∣*k*) are predicted performance and power at time *k* + *j*; *Q*_*j*_ and *R*_*j*_ are the weights(matrices if *perf*(*k* + *j*∣*k*) and *power*(*k* + *j*∣*k*) are vectors) between performance and power. Note that here we assume that there is only one PM and omit the subscript *i* in *S*_*i*_(*k*), *perf*_*i*_(*k*), *power*_*i*_(*k*).

In Eq (5), the first part represents the quality of performance. $\hat{\lambda }(k+j)$, the prediction of workload, is the required performance at time *k* + *j*. Because of time delay and uncertainty, it is possible that current control cannot create the desired effect immediately. Therefore, the difference $\mathrm{perf}(k+j|k)-\hat{\lambda }(k+j)$ can be treated as performance tracking error. And the quadratic term $\|perf(k+j|k)-\hat{\lambda }(k+j){\|}_{Q_{j}}^{2}$ is the error with weight *Q*_*j*_. $\|power(k+j|k){\|}_{R_{j}}^{2}$ is the power value with its weight *R*_*j*_. Here *power*(*k* + *j*∣*k*) contains the switching cost caused by time delay. For example, the idle power is actually included in Eq (5). The larger *Q*_*j*_ and smaller *R*_*j*_ mean that the controller cares performance more, and vice versa. Obviously, appropriate choice of *Q*_*j*_ and *R*_*j*_ is significant for control, otherwise it cannot lead to a good balance between performance and power. This point will be discussed in Section 5 with more details.

The predictive controller aims to minimize the cost function at each time while state transition graph defines constraints for control action *u*(*k* + *j*):

$$\begin{array}{l} \underset{\left\{u\left(k+1\right),..,u\left(k+P\right)\right\}}{\min}\;cost\left(k\right),\\ \text{subject}\;\text{to}\;\text{that}\;u\left(k+j\right),j\;=\;1,\ldots ,P,\;\text{coincides}\;Fig.3 \end{array}$$ (6)

and it works as below. At each time *k*, the system state *S*(*k*) now is known. Then by choosing control action sequence *u*(*k* + 1), …, *u*(*k* + *P*), a predictive controller can predict the virtual state path $\hat{S}(k+1),...,\hat{S}(k+P)$ in the future *P* steps according to the state model, i.e. Fig 3. Different control sequence *u*(*k* + 1), …, *u*(*k* + *P*) will lead to different state path $\hat{S}(k+1),...,\hat{S}(k+P)$, which will lead to different performance and power prediction too. Given the cost function defined above, the optimal state path can be found and thus the optimal control sequence can be determined. Then, the optimal control action *u*(*k* + 1), …, *u*(*k* + *M*), *M* ≤ *P*, in the future *M* steps can be determined, which will be adopted by *PM* at time *k*.

When there are more jobs and more machines, performance and power in Eq (5) can be defined as a vector. Then *Q*_*j*_ and *R*_*j*_ are weight matrices and the element implies the weight between each job and each machine, which makes the calculation of *cost*(*k*) more complicated. However, the predictive controller can be designed in a similar way.

### 4.2 Prediction for Workload

From description above, prediction of future workload $\hat{\lambda }(k+j)$ is needed for the controller to optimize the cost function. Here we assume that workload cannot wait and accumulate, thus its prediction is independent of controller. There are certain statistical models in the literature, in which the typical ones are ANOVA and AR model, or models combined with them. We refer to [17] for more details.

Briefly speaking, ANOVA model is suitable to analyze data for long time which have repeatable pattern, while AR model is suitable for prediction of data for shorter time. Since we only need the instant prediction and do not focus on data’s pattern, we will apply the AR(Auto-Regression) model to predict workload in this paper.

For a sequence {*x*(*k*)}, an AR model with order *r*, simplified as AR(r) model, is to utilize linear combination of history data to fit the sequence and predict future values, i.e., to assume that

$$\begin{array}{c} \hat{x}\left(k\right)=\overset{r}{\underset{i=1}{\sum }}\;a_{i}\cdot x\left(k-i\right)+e_{k} \end{array}$$

where *e*_*k*_ is noise and parameters *a*_*i*_ can be estimated off line or online.

By Eq (5), at each time *k*, we need to get the prediction $\hat{\lambda }(k+1),...,\hat{\lambda }(k+P)$. Take *r* = 2 in the model as an example. Then we have

$$\begin{array}{c} \hat{\lambda }\left(k\right)=a_{1}\cdot \lambda \left(k-1\right)+a_{2}\cdot \lambda \left(k-2\right). \end{array}$$

The parameters *a*_1_, *a*_2_ can be estimated by data fitting offline from history data. Then, at time *k* + 1, suppose we have *λ*(1), …*λ*(*k*), predictions for the future *P* steps are obtained by

$$\begin{array}{ll} \hat{\lambda }\left(k+1\right) & =a_{1}\cdot \lambda \left(k\right)+a_{2}\cdot \lambda \left(k-1\right);\\ \hat{\lambda }\left(k+2\right) & =a_{1}\cdot \hat{\lambda }\left(k+1\right)+a_{2}\cdot \lambda \left(k\right);\\ ...... & \\ \hat{\lambda }\left(k+P\right) & =a_{1}\cdot \hat{\lambda }\left(k+P-1\right)+a_{2}\cdot \hat{\lambda }\left(k+P-2\right) \end{array}$$ (7)

in the order.

### 4.3 Optimization

At time *k*, suppose that we have predicted the future workload, we are at the place to choose control action sequence *u*(*k* + 1), …, *u*(*k* + *P*) to solve the Eq (6), i.e., to optimize *cost*(*k*). Still the workload is assumed to be abandoned if it is not dealt with at current time. Note that the optimization can not be finished directly by a Matlab function, as [18] did.

Due to the discrete state model, choice of *u*(*k* + 1), …, *u*(*k* + *P*) seems a tough task. It is true for the worst case when the state transition graph Fig 3 is a complete directed graph, i.e., all the states can be transited to all the states in one step. At this case, the state of a single PM can be taken from 6 different values, so the state space for *P* times has a volume of 6^*P*^, which will increase exponentially with state number and thus will be very huge when there are many states of a PM or CPU.

However, in the scenario of this paper, the state transition must satisfy physical constraints, which causes that Fig 3 is not a complete digraph and *u*(*k* + 1) can only be transited to certain states. This will shrink the state space greatly. On the other hand, considering the performance requirement in practice, the control actions which are far from being able to satisfy performance requirement, will also be abandoned first.

In this paper, we will search on the state space in the future *P* times. Then performance and power and thus the cost function *cost*(*k*) for each state path in the prediction horizon can be calculated according to the model. By choosing the path with minimal cost function, we can determine the control actions for the future *M* times.

**Remark 4.1:** For optimization Eq (6), another feasible sophisticated approach is to use dynamic programming. To do this, we need to draw the state transition graph for future *P* steps originating from current state, which is actually a tree. When the state space is huge and *P* is big, dynamic programming might be computationally efficient.

**Remark 4.2:** For large scale computing systems covering a large number of processors, e.g., more than 1000, the state space will amplify sharply which will result in great challenge on search algorithms. To deal with this, the usual method is to compute off line based on history data. On the other hand, there are often several batches of homogeneous machines in real systems, which can also simplify the problem highly by coarse grained modelling. Surely this brings about another tradeoff between coarse grained and finer grained modelling, which actually is a tradeoff between high-cost-optimal-outcome and low-cost-suboptimal-outcome.

## 5 Experiment Validation

In this section, experiments will be implemented to check the effectiveness of the predictive controller based on the discrete state model.

### 5.1 Test Bed

We will take *n* = 3, *m* = 2, i.e., the system is composed of 3 physical machines *PM*_1_, *PM*_2_, *PM*_3_, and at most 2 virtual machines can be hosted on each physical machine. Configuration of the PMs are listed in Table 1.

**Table 1:** Configuration of physical machines.

**PM name**	**Core/thread**	**DVFS frequency**	**Idle power(w)**	**Max power(w)**
*PM*_3_ (s-i7)	4/8	1.60, 2.93GHz	177	229
*PM*_2_ (s-cf)	4/4	1.99, 2.49GHz	138	178
*PM*_1_ (s-quad)	4/4	2.00, 2.83GHz	115	176

The processors of *PM*_1_, *PM*_2_, *PM*_3_ are Core i7, Xeon 5320, Core 2 Quad respectively, the OS is Fedora 16, and the virtualization software is KVM. Note that these PMs or processors are chosen to be different, which can happen usually in practice. The heterogeneity on PMs or processors can be easily handled in the experiment by choosing appropriate parameters in performance and power models; we will see that they will not influence the effectiveness of our controller, which implies extendibility of the method.

The parameters pertinent to performance and power during control process are measured from chosen servers and listed in Table 2 below, in which it is set 1*T* = 5 *min*, and perf, freq are abbreviations for performance, frequency.

**Table 2:** Parameters of control actions about performance and power.

**Possible control action**	**Time delay**	**Power**	**Perf delay**
turning off a PM	1T	max power	0
turning on a PM	2T	max power	0
changing freq by DVFS	0	determined by current freq	determined by new freq
turning on a VM	1T	power after VM turning on	0
turning off a VM	0	power before VM turning down	0
VM immigration	0	determined by new PM	determined by new PM

**Fig 4:**
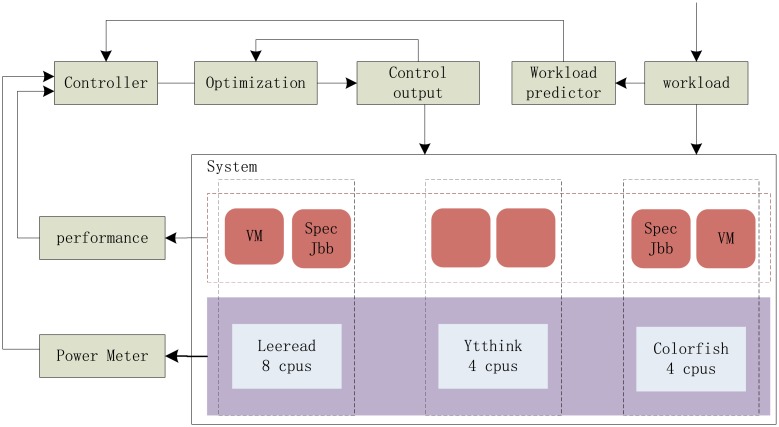
The architecture and control flow in the virtualized computing system.

Then the flow chart with predictive controller in the virtualized computer system is drawn in Fig 4.

The data of workload in our experiment can be seen in S1 File.

### 5.2 Choice of Control Parameters

By the definition of *cost*(*k*) in Eq (5), four parameters will influence its value: the integers *P*, *M* and the weights *Q*, *R*. Hence, they will influence the solution of optimization Eq (6) and thus the effect of controller. Additionally, *P*, *M* influence complexity of the problem more heavily while *Q*, *R* will influence the choice of control actions more significantly.

Now, we decide the prediction and control horizon *P* and *M* first. As we have stated above, long prediction horizon will lead to exponentially increasing search space for predicted state, which is not bearable for large scale servers. So *P* cannot be very large. On the other hand, *P* must be chosen according to the characteristics of workload so that prediction accuracy can be assured. With deeper prediction steps, prediction of workload will become less accurate. Based on these points, we take *P* = 3 in the experiment. *M* is taken as 1 as in the literature to avoid the conflict between control actions at time *k* and *k* + 1.

Then, we decide the weights *Q*_*j*_ and *R*_*j*_. First, we set *Q*_*j*_ = 1, *j* = 1, 2, …, *P*, by which we mean that the cost resulting from performance tracking at each time are the same.

As to the power saving weight *R* (denoted by *R* since *M* = 1), it is important to choose a moderate value for it: different *R* leads to different cost function and thus different optimal control action, and then different performance quality and power consumption. Moreover, it can be imagined that a moderate value of *R* is dependent of jobs’ types. In the experiment, we will choose different *R* for an identical workload sequence, and compare the results under each *R*.

### 5.3 Experiment Results

To test the effectiveness of predictive controller, we will compare performance and power of the system under control with the case without controller(or equally, with open loop controller). For the system with predictive controller, we will compare experiment results under different choice of *R*.

When the system is managed without controller, all physical machines will be always turned on so as to guarantee the performance requirement, thus turning on/off a PM is not an option of control actions in this case. This is just the practice for many data centers today.

We define several criteria as below:

$$\begin{array}{l} PoU=\frac{\left|\{k:\;perf\left(k\right)<\lambda \left(k\right)\}\right|}{\left|k\right|}\cdot 100\%,\\ NNR=mean\left(\left(\lambda \left(k\right)-perf\left(k\right)\right)\cdot 1_{\left\{perf\left(k\right)<\lambda \left(k\right)\right\}}\right),\\ PoS=\left(1-\frac{\sum_{k}power_{olc}\left(k\right)}{\sum_{k}power_{pc}\left(k\right)}\right)\cdot 100\%, \end{array}$$ (8)

where *mean*(*x*(*k*)) is average of sequence *x*(*k*), *power*_*olc*_(*k*) is the measured power without controller, and *power*_*pc*_(*k*) is the measured power with predictive controller. It is easy to see that *PoU* stands for the *percent* of times *under* performance requirement(i.e., the performance is not satisfied), *NNR* stands for the *number* of *non-handled-request*, and *PoS* stands for the *percent* of power *saving* compared with the open-loop-controller case.

#### 5.3.1 Basic Results

Table 3 below roughly shows the experiments results with different *R*:

**Table 3:** Performance and power criteria under different *R*.

*R*	*PoU*	*NNR*	*PoS*
5	0.23%	1000	21%
50	0.24%	3478	33%
100	38.00%	7911	35%
200	50.20%	11854	38%
500	74.48%	35582	51%

From Table 3, we can see that the predictive controller can save power considerably compared to the system with open-loop controller while the performance tracking result is very different under different *R*. To be specific,

(1). When *R* = 5, *PoU* is very small, being 0.23%, meaning that the performance requirement can be satisfied for almost all the times. Averagely, 1000 requests cannot be handled at each time. However, in this case, the power can be saved only by 21%, which is not bad but can be improved still.

(2). When *R* = 50, *PoU* is also very small, being 0.24%, which is almost the same with *R* = 5 case. And now there are 3478 requests which will be abandoned at each time. On the other hand, power consumption can be saved by 33%, which is great progress to *R* = 5 case.

(3). When *R* = 100, *PoU* now increases pretty highly, being 38%, implying that for more than one-third time, the performance requirement cannot be satisfied, which is very bad. And now the amount of requests that cannot be handled at each time is high, being 7911. At the same time, power is saved by only 35%, which is very near that of *R* = 50 case.

(4). When *R* = 200, *PoU* now becomes 50.2%, implying that performance cannot be satisfied for more than half of the time, while power is saved by 38%, which is still near that of *R* = 50 case.

(5). When *R* = 500, *PoU* now becomes 74.48%, implying a terrible performance. Power consumption is saved by 51%, which is a great progress to smaller *R*. Obviously, we cannot choose such a decision since performance is prior to power.

With respect to increasing *R*, results in Table 3 coincide with the physical meaning of *R*: with *R* increasing, power saving holds a bigger weight in the cost function, and thus power saving can become better, while performance becomes worse. We can find that *R* = 50 is a suitable weight, which can achieve a good balance between performance tracking (99.76% requirement can be deal with) and power saving (energy consumption can be saved by 33%).

#### 5.3.2 Control Process in Details

**Fig 5:**
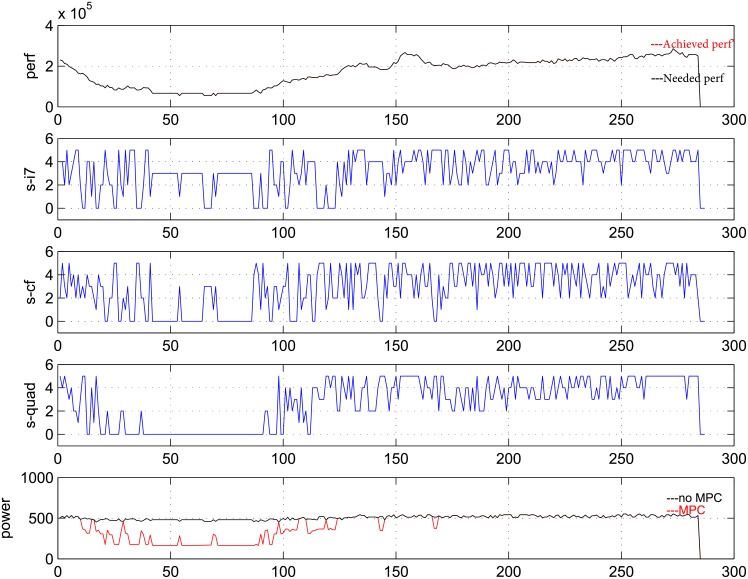
Performance, power and state transition in one day at R = 5.

**Fig 6:**
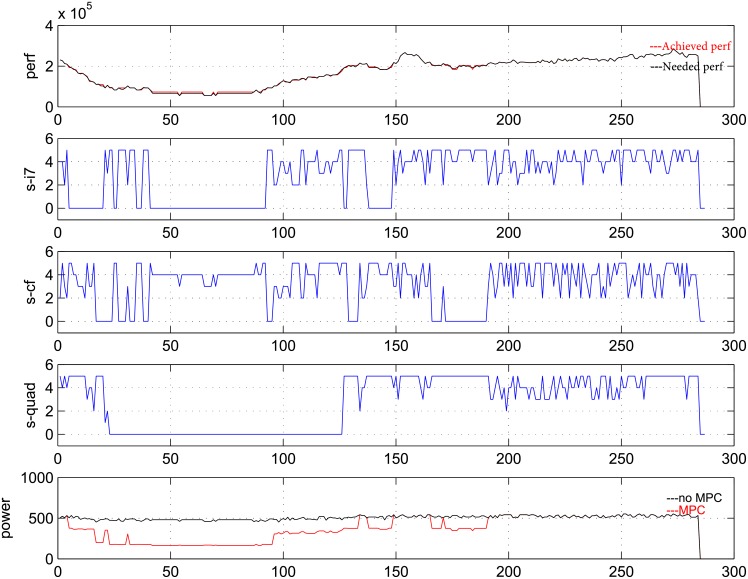
Performance, power and state transition in one day at R = 50.

**Fig 7:**
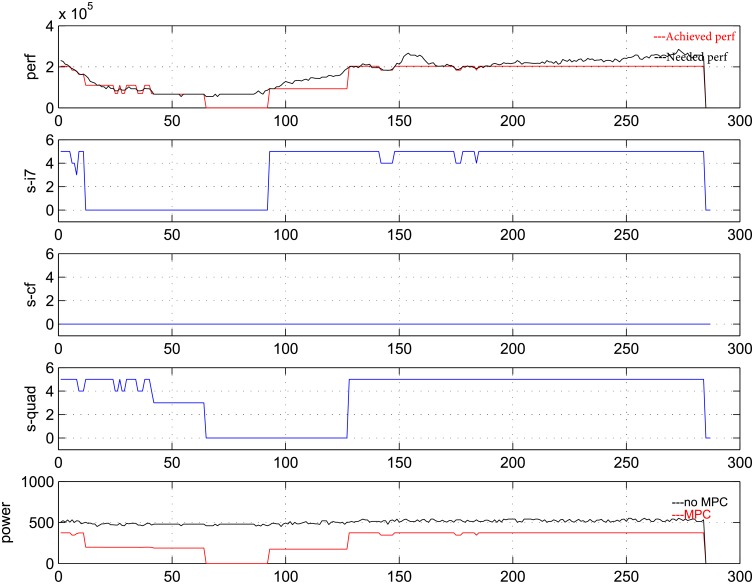
Performance, power and state transition in one day at R = 500.

Next, we will present the detailed process of the predictive controller. To this end, curves of the state, performance and power for three PMs are shown in each of Fig 5, Fig 6, Fig 7 as below, which contains five pictures in them. The data is measured for 24 hours every 5 minutes and the weight *R* is taken as 5, 50, 500 respectively in three figures.

In Fig 5, Fig 6, Fig 7, the horizontal axis denotes time; the vertical axis of the first picture denotes number of requirements that are coming and handled; the vertical axes of the second, third and fourth pictures denote the frequencies of three PMs; the vertical axis of the fifth picture denotes total power of the system. In the first picture, black curve denotes the coming requirement while red curve denotes the handle requirement at each time. In the fifth curve, black curve denotes the power without controller while red curve denotes the power with predictive controller.

From the curves, control actions can be distinguished from the change of frequencies and power clearly. When the frequency is zero and the power is smaller than the sum of idle powers, we can see that PM under observation is indeed shut down. And we can also see that under open loop control, the power is always maximal.

From Fig 5, Fig 6, Fig 7, when *R* = 5, the action of turning on/off the PM is used rarely, and the performance can be satisfied well while power saving is not very good. When *R* = 500, one PM is always being turned off, the power saving is good, while the performance tracking is bad. *R* = 50 is a moderate choice, which can lead to a good balance between performance and power. Meanwhile, at *R* = 50, VMs can be migrated between PMs, and power cycling is an effective control action.

To sum up, the choice of *R* is very crucial for control. If *R* can be taken moderately, such as *R* = 50, then a good balance between performance and power can be achieved: on one hand, the performance can be satisfied well; on the other hand, the power consumption can be saved considerably. In practice, *R* can be obtained by learning from experience, which depends on the specific system and jobs.

## 6 Conclusions and Future Work

In this paper, the power saving problem in the nonlinear virtualized computing system is studied. First, we present some novel nonlinear characteristics of newer servers which are found from data. Such nonlinear features make linear power model not suitable again for fine control. Then, we build a discrete system state model, in which all control actions such as turning on/off a PM (or a VM) can be included and time delay effect can be reflected too. Then, by defining a quadratic cost function which involves both performance and power, the predictive controller based on the discrete state model can be designed in a natural way. Thus the cost function can be optimized by regulating computing resources dynamically. Experimental results show that with an appropriate weight of performance and power in the cost function, the predictive controller can achieve efficient performance and power management: almost 99.76% requirements can be dealt with and 33% power consumption can be saved compared with the case without controller.

In practice, the architecture of processors is also important and needs to be taken into account. Additionally, as we have noted before, the proposed discrete state model in this paper is still very rough. In order to get better effect, more sophisticated models need to be built so that more details can be included such as the share of VCPU, the frequency values of processors and so on. These will be left in the future work.

Finally, new technologies have been booming in control theory, such as the theory on multi-agent systems, in which massive agents with individual dynamics are contained and the consensus or special formations are often control objectives([26][27]). It can be surprisingly correlated with distributed computation, see [28][29]. Will such theory provide some inspirations to problems under study in the current paper? We do not know now while it might be interesting if new relations can be built.

## Supporting Information

S1 File (XLS)
